# Work motivation and associated factors among nurses working in public hospitals of the Wolaita Zone, Southern Ethiopia

**DOI:** 10.3389/frhs.2025.1561643

**Published:** 2025-07-10

**Authors:** Bereket Dejene Senbetu, Wadu Wolancho Debancho, Tsiyon Mekoya Jemaneh, Serkalem Aschalew Jima, Temesgen Geta Hardido, Tamirat Beyene Gerete

**Affiliations:** ^1^ School of Nursing, College of Health Sciences and Medicine, Wolaita Sodo University, Wolaita Sodo, Ethiopia; ^2^ School of Nursing, Institute of Health Sciences, Jimma University, Jimma, Ethiopia; ^3^ Department of Nursing, College of Health Sciences, Arsi University, Asella, Ethiopia; ^4^ School of Midwifery, College of Health Sciences and Medicine, Wolaita Sodo University, Wolaita Sodo, Ethiopia

**Keywords:** nurses, work motivation, health professionals, public hospitals, Wolaita Zone

## Abstract

**Background:**

Nurses’ work motivation encompasses their readiness to perform effectively and achieve designated tasks and objectives. Providing high-quality nursing care becomes significantly more challenging in the absence of motivated nurses and a supportive healthcare environment. Nevertheless, there is a paucity of empirical research examining the work motivation of nurses. Hence, this study aimed to assess the level of work motivation and its associated factors among nurses working in public hospitals in the Wolaita Zone.

**Methods:**

An institutional-based cross-sectional study involving 419 nurses and 12 key informants was conducted using a mixed-methods method. Quantitative data were collected using a simple random sampling technique, while qualitative data were collected using purposive sampling. Data were collected using pretested self-administered questionnaires and in-depth interviews. Statistical Package for Social Science version 26 was used for statistical analysis. Bivariate and multivariable logistic regression analyses were performed to identify factors associated with work motivation. Statistical significance was determined using AORs with 95% CIs and *p*-values of less than 0.05.

**Results:**

A total of 394 participants were included, yielding a response rate of 94%. About 202 nurses (51.3%; 95%CI: 46.4–56.9) reported being motivated. Several factors were negatively associated with nurses’ work motivation: type of institution (AOR = 0.406; 95% CI: 0.236–0.699; *p* = 0.001), workload (AOR = 0.495; 95% CI: 00.297–0.827; *p* = 0.007), negative perceptions of respect and benefits for nurses in organizations (AOR = 0.351; 95% CI: 0.180–0.682; *p* = 0.002), negative perceptions of leadership in hospitals [(AOR = 0.487; 95% CI: 0.240,0.985; *p* = 0.045). In contrast, nurses with 1–5 years of work experience were likely to be motivated (AOR = 3.29; 95% CI: 1.101–9.846; *p* = 0.003). The analysis revealed themes related to structural-administrative factors, socio-economic factors, and individual nurse-related factors.

**Conclusion:**

The results reveal that approximately 50% of the nursing participants expressed low levels of work motivation. Significant factors influencing nurses’ motivation included the type of institution, years of work experience, workload, perceptions of respect and benefits, and quality of leadership in hospitals.

## Introduction

Worker motivation arises from the dynamics between individuals and their work settings, as well as how well these dynamics align with the larger societal framework (1).

**Table 1:** Sample size determination considering factors associated with nurses’ work motivation, August 2023.

Variables	Power	Ratio	%outcome in exposed	%outcome in unexposed	CI	RR	OR	n+10% NRR	Final sample size	Source
Qualification	80	1	46.32	64.1	95%	1.38	2.0	266 + 26	292	(26)
Experience	80	1	35	58.3	95%	0.6	0.38	160 + 16	176	(26)
Perceived respect for nurses	80	1	41	62	95%	0.66	0.42	196 + 19	215	(26)

There are two different types of motivation: intrinsic and extrinsic. Intrinsic motivation happens when a person does a task or activity for its own sake and favor, driven by personal interest and the pleasure derived from doing the task. Extrinsic motivation involves doing a task or activity for instrumental explanations (2, 3). Healthcare professionals are the most valuable resource within the healthcare system; institutions that prioritize the enhancement of knowledge and skills, foster motivation, and create opportunities for professional development are generally more effective and successful (4). According to the latest WHO report, about 27 million people worldwide work in the nursing and midwifery field (5). Nurses, as a significant component of hospital human resources, comprise 60%–70% of the total hospital workforce (6). As reported by the Ethiopian Nursing Association (ENA) and MOH, more than 43,500 nurses are working in the government system, equating to one nurse for every 1,705 people and one midwife for every 5,491 people (7–9). In Ethiopia, research indicates that management practices and the working environment are not conducive to delivering high-quality nursing care, which subsequently impacts nurses’ performance and the quality of care provided (9).

A health system cannot achieve its desired outcomes without motivated healthcare providers. Nurses play a vital role in healthcare by delivering efficient treatment, identifying effective interventions, and facilitating health emergencies, acting as bridges between patients and the healthcare team. High-quality nursing care ensures that healthcare needs are met while prioritizing patient safety, dignity, and comfort. This type of care is also characterized by consistency, positive patient outcomes, and high levels of patient satisfaction (10, 11).

Health system sector reforms are basically ongoing processes of change that can add extra destabilization to the working environment; these reforms aim to strengthen national strategies, policies, and programs by altering healthcare priorities, regulations, policies, structure, and financing systems (12).

Numerous global studies have examined the work motivation of nurses, identifying motivation and job satisfaction as critical determinants of retention and turnover among nurses and healthcare professionals in low- and middle-income countries (13, 14). While job satisfaction in nursing significantly influences the likelihood of leaving the profession or experiencing burnout, insufficient work motivation adversely impacts nurses’ performance and engagement (6, 15).

According to the WHO report, there is a shortage of nurses and midwives, which has been a big challenge for the last 10 years in many countries. To reach SDG-3 on health and well-being, an additional 9 million nurses will be needed by 2030. This goal can only be achieved if there are enough nurses who are well-trained, educated, regulated, and properly supported. Moreover, nurses should be well paid and recognized according to the quality of care they provide (5, 16). Nurses’ performance significantly affects the quality of care they provide, with higher levels of motivation leading to better performance and achievement of the vision, mission, and goals of healthcare institutions (17, 18). Conversely, reduced motivation among nurses adversely impacts the quality of care, leading to a higher likelihood of patients seeking alternative healthcare options and contributing to rising medical expenses (19).

A scoping review of nursing motivation identified that encouragement of autonomous motivation may lead to an increase in work engagement while decreasing workaholism and burnout (20). The association between low motivation and burnout is underscored by a systematic review, which indicated that approximately 39% of Ethiopian nurses express intentions to leave the profession (7). Additionally, the impact of low motivation on the quality of care is evident, as another systematic review revealed that a significant proportion of hospitalized patients reported dissatisfaction with the nursing care they received (21). In Ethiopia, the problem of nurses’ work motivation has not been sufficiently studied. Studies conducted among healthcare professionals, including nurses, reported varying levels of motivation across different regions, ranging from as low as 19.5% to as high as 63.63% (22–26). A recent study conducted across four regions among health extension workers and other health professionals reported a motivation level of 61%, with significant variations observed across the regions (27). This indicates differences in study design, population size, settings, and the tools used to measure motivation. To better study the problem of motivation, it is important to focus exclusively on nursing professionals rather than on healthcare professionals. Generally, evidence from different studies suggests that low job satisfaction and poor motivation are the leading causes of nurse attrition and turnover intentions (14, 28, 29). The WHO has highlighted work motivation as one of the key factors for resolving problems relating to the recruitment and retention of staff in the healthcare sector (30). However, this issue is a great concern for nurses in Ethiopia because there is a high turnover rate among nursing professionals (31).

Various organizational and personal factors influence nurses’ work motivation, leading to dissatisfaction and a desire for a more supportive work environment (32). These factors collectively affect nursing management and performance, leading to detrimental outcomes for nurses and, ultimately, posing serious risks to patient safety (33–35).

As the literature suggests, the level of motivation and the factors affecting it vary over time and across institutions. In Ethiopia, while some studies have explored the same topic among healthcare professionals in general, few have focused on nurses. Based on recommendations from previous studies, involving key informants, such as nursing managers and administrators, is significant for achieving the study objective. To the best of the researcher’s knowledge, such research is limited, and not much was explored in a particular study area. Therefore, this study intends to assess the level of work motivation and its associated factors among nurses working in public hospitals in the Wolaita Zone.

## Methods and materials

### Study design, period, and setting

An institution-based cross-sectional mixed-method study was conducted from June 1 to 30, 2023. The Wolaita Zone has a total area of 4,511.7 km^2^. Based on the projections of the 2007 Population and Housing Census, the population of the Wolaita Zone was approximately 2,161,842 in 2014 E.C. There are nine governmental hospitals (one comprehensive specialized hospital and eight governmental primary hospitals) and 68 health centers in the Wolaita Zone. A total of 1,656 nurses are employed in the Wolaita Zone (36).

### Study population and eligibility criteria

All randomly selected nurses available during the study period in public hospitals in the Wolaita Zone were included for the quantitative data collection. All purposively selected nurses, nurse managers, and leaders available during the study period in public hospitals in the Wolaita Zone were included for the qualitative data collection. Nurses who provided unpaid service and were employed on a part-time basis in each hospital were excluded.

### Sample size calculation and sampling procedure

The sample size was calculated using a single population proportion formula, based on the motivation level from a previous study of 54.5% (*p* = 0.545) conducted in Jimma Town, southwestern Ethiopia (26), with a 5% marginal error (d) and a 95% confidence interval (*Z α*/2 = 1.96) from the total number of nurses in public hospitals in the Wolaita Zone. Based on these assumptions, the sample size is calculated as follows:

$$n=\frac{{\left(z_{\alpha /2}\right)}^{2}p(1-P)}{d^{2}}$$

where *n* is the minimum sample size required, *p* is the estimated proportion of motivation, which is 54.5% (0.545), *Z_α_*_/2_ is the *Z*-score corresponding to a 95% significance level (*α* = 0.05), which is  = 1.96, and *d* is the margin of error to be tolerated, which is set at 0.05 (5%)

$$n=\frac{{\left(z_{\alpha /2}\right)}^{2}p(1-P)}{d^{2}}$$

$$n=\frac{{\left(1.96\right)}^{2}(0.545)\;(1-\;0.545)}{{\left(0.05\right)}^{2}}=\frac{{\left(1.96\right)}^{2}\times 0.545(0.455)}{{\left(0.05\right)}^{2}}=\frac{3.8416\times 0.248}{0.0025}=\frac{0.9526}{0.0025}=n=381$$

Then, considering a 10% non-response rate, 38 additional participants were added to the initial sample size of 381, resulting in a final sample size of 419 (381 + 38), i.e., *n* = 419.

The final total sample size was 419. Therefore, 419 nurses working in selected public hospitals of the Wolaita Zone were included as study subjects.

The sample size for the second objective (i.e., identifying factors associated with nurses’ work motivation) was calculated using Epi Info software 7.2.6 by considering a confidence level of 95%, a margin of error of 5%, a power of 80%, and a ratio of 1:1 (see Table 1).

However, the sample size calculated using the single population proportion (i.e., proportion of nurses’ motivation) was larger than that calculated for the predictor variables. Therefore, the sample size derived from the single population proportion formula (i.e., proportion of nurses’ motivation), which is 419, was taken as the sample size for this study.

Twelve key informants were purposely selected from nurses working in public hospitals in the Wolaita Zone for conducting in-depth interviews for qualitative data collection. The number of key informants was determined based on the saturation of the required data.

Proportional sampling methods were applied across all public hospitals. A comprehensive list of nurses employed at each facility was compiled to serve as the sampling frame. From each hospital, nurses were then chosen utilizing a simple random sampling approach facilitated by a computer-generated process in Microsoft Excel. Subsequently, eligible individuals from each public hospital were recruited to participate in the study. For the qualitative data component, criterion-based purposive sampling was employed to identify key informants for in-depth interviews. Specifically, selected nurses, along with nursing leaders and managers, were designated as participants in this study.

### Study variables

Nurses' work motivation was the outcome variable of this study. The independent variables included socio-demographic factors (age, sex, marital status, years of service, income, educational qualification), organizational/institutional factors (type of institution, training and skill development, leadership quality in the hospital, respect for nurses, opportunities, achievement/goal clarity, remunerations/incentives), and work environment factors (interpersonal relationships, workload, availability of resources, work unit, and position at work).

### Operational definitions

**Motivated**: Nurses were classified as motivated if their total motivational score was greater than or equal to the mean score of 117.43 (26).

**Unmotivated**: Nurses were classified as unmotivated if their total motivational score was less than the mean score of 117.43 (26).

**Intrinsic motivation**: A type of motivation that happens when a person does a task or activity for its own sake and favor, driven by personal interest and the pleasure derived from doing the task (2).

**Extrinsic motivation**: A type of motivation that refers to doing a task or activity for instrumental reasons, for instance, receiving incentives, recognition, alleviating penalties, gaining good comments, and appreciation, which contribute to improving self-esteem and directing efforts toward achieving specific targets (3).

**Job satisfaction:** It represents a positive emotional response an individual experiences toward their work, as long as their professional values are respected (37).

### Data collection tools and techniques

The data collection tools consisted of close-ended questionnaires and interview guides, which were adapted from previously validated tools (26, 38–40). The self-administered questionnaires were prepared in English to collect the data. The assessment tools consisted of three parts: socio-demographic characteristics, organization-related questions, and motivation measurement questions, which included multiple items and sub-items assessed using a Likert scale. Motivation was measured using different categories of motivation measurement items based on the Likert scale. All statements were scored on a five-point Likert scale (strongly agree = 5, agree = 4, uncertain = 3, disagree = 2, and strongly disagree = 1). The items included in the motivation measurement scale included the following: satisfaction, with four questions; benefit, with four questions; recognition, with three questions; supervision, with four questions; management and administration, with eleven questions; achievement/goal clarity, with four questions; and opportunities, with four questions (26).

### Qualitative data collection

A semi-structured interview guide with open-ended questions was employed to collect qualitative data. The in-depth interview questions were translated into the local language to facilitate data collection. In alignment with the research objectives, probing and follow-up inquiries were utilized to achieve a comprehensive understanding of the study's subject matter. Consequently, the interview guides were used to investigate perspectives, opinions, and recommendations that were not captured during the quantitative data collection phase. Ultimately, the gathered data were organized into specific thematic categories.

Data collection was conducted at each hospital using a self-administered questionnaire. Three BSc nurses were selected and trained specifically for this purpose. The data collection process was supervised by a supervisor and the principal investigator. Qualitative data were obtained through face-to-face interviews with purposefully chosen participants, utilizing a semi-structured in-depth interview guide. Interviews were documented using field notes and audio recordings.

### Data quality assurance

Two days of training was provided to data collectors and supervisors, focusing on the content of tools and the rights of participants. The investigators and supervisors monitored the data collection process daily for completeness, accuracy, clarity, and consistency. All data were double-entered and cleaned before analysis. To ensure clarity, comprehension, and flow of questions, the questionnaire was pretested on 5% (20 nurses) of the sample population at Shone Primary Hospital 1 week before the actual data collection began. Appropriate corrections were made, including the logical order of some questions, revision of difficult-to-understand words, and the removal of some items. The reliability of the motivation measurement scale and the organizational/work environment-related questions was checked for internal consistency using Cronbach's alpha, yielding coefficients of 0.97 and 0.76, respectively. Subject area experts checked content validity.

To ensure the quality of the qualitative data, the audio recordings were attentively listened for insights, and the field notes were extensively rechecked and repeatedly read.

### Data processing and analysis

Following data collection, the collected data were manually checked for completeness by the supervisor and researcher and then entered into EpiData version 4.6. Afterward, the data were exported to SPSS (Statistical Package for Social Sciences) version 26 for further cleaning, coding, and analysis. Descriptive statistics were used to describe each variable using frequencies, percentages, means, and standard deviations. Variables with a *p*-value of less than or equal to 0.25 in the bivariate logistic regression analysis were included in the multiple logistic regression analysis to control for potential confounders. Adjusted odds ratios (AORs) with 95% CIs were used to identify predictor variables. Variables with a *p*-value <0.05 were considered statistically significant.

Results were summarized and presented using narrative descriptions, graphs, and tables. Multicollinearity was checked to assess the linear correlation among the independent variables using the variance inflation factor (VIF), tolerance, and standard error. None of the variables yielded variance inflation factor >10, tolerance <0.1, and standard error > 2 (VIF <1.862, tolerance >0.537, and std. error <0.048); therefore, no variables were excluded from multivariable analyses. The Hosmer and Lemeshow test was found to be insignificant (*p*-value = 0.139) and the Omnibus test was significant (*p*-value = 0.000), indicating that the model was fitted.

Audio recordings in Amharic were transcribed and translated into English. Two authors coded the data segment by segment using open coding with the support of Atlas.ti version 7.5, following an extensive reading and re-reading of the transcripts. Then, codes were organized into categories, and themes were triangulated with the quantitative findings for discussion. The findings were quoted and narrated by mentioning the interviewees’ IDs and types.

### Ethical approval

This research was conducted in accordance with the Declaration of Helsinki. Ethical approval was obtained from the Ethical Review Committee of Jimma University (Ref. No.: JUIH/IRB/398/23). Subsequently, a letter was submitted to the administration of each hospital to obtain permission before data collection. Before data collection, ethical clearance was obtained from the institutional review board of Jimma University, Institute of Health, and submitted to each hospital. Permission letters were obtained from each hospital before data collection. Participants were informed that their answers to the questions would be grouped with those of other respondents and reported as part of the research study.

## Results

### Socio-demographic characteristics of nurses

Out of 419 individuals approached, 394 completed the questionnaire, resulting in a response rate of 94%. Among the participants, 239 (59%) were women. The mean age of the respondents was 33.07 years, with a standard deviation of 4.51 years, with 230 participants (58%) falling within the age range of 31–40 years. Regarding religious affiliation, 184 participants (46.7%) identified as Protestant, while 286 respondents (72.6%) belonged to the Wolaita ethnic group. Additionally, 206 participants (52.3%) reported being married (Table 2).

**Table 2:** Socio-demographic characteristics of nurses working in public hospitals in the Wolaita Zone, southern Ethiopia, August 2023 (*n* = 394).

S. no.	Characteristics	Category	Frequency	Percent
1	Sex	Female	223	56.6
		Male	171	43.4
2	Age, years	20–24	2	0.5
		25–29	114	29
		30–34	144	36.5
		35–39	85	21.6
		≥40	49	12.4
3	Ethnicity	Wolayta	286	72.6
		Oromo	34	8.6
		Amhara	47	12
		Tigre	10	2.5
		Others^b^	17	4.3
4	Religion	Protestant	184	46.7
		Orthodox	171	43.4
		Muslim	20	5.1
		Others^a^	19	4.8
5	Marital status	Married	206	52.3
		Single	181	45.9
		Divorced	5	1.3
		Widowed	2	0.5
6	Educational level	Diploma	61	15.5
		BSc	310	78.7
		MSc	23	5.8
7	Position in work	Staff nurse	345	87.6
		Nursing leaders	49	12.4
8	Registered in the nursing profession, years	1–5	191	48.5
		6–10	168	42.6
		>11	35	8.9
9	Work experience, years	1–5	218	55.3
		6–10	168	42.7
		>11	8	2
10	Monthly salary, ETB	<3,000	2	0.5
		3,001–6,000	179	45.4
		>6,000	213	54.1
		General ward	142	36
		Emergency ward	93	23.6
		Critical care unit	18	4.6
		OPD	91	23.1
		Oby/gynecology ward	19	4.8
		Operation room	14	3.5
		Other^c^	17	4.3

^a^ Hawariyat, Adventist, or Catholic.

^b^ Hadiya, Gamo, or Dawuro.

^c^ ART Clinic, Optha, PACU.

**Table 3:** Socio-demographic characteristics of qualitative study (IDI) participants in public hospitals in the Wolaita Zone, southern Ethiopia, August 2023 (*n* = 12).

Participants	Code	Age	Sex	Educational status	Working experience	Position
1.	P1IDI	33	F	BSc	8 years	Nursing director (matron)
2.	P2IDI	34	F	MSc	7 years	Staff nurse
3.	P3IDI	36	M	BSc	4 years	Nursing director (matron)
4.	P4IDI	32	M	BSc	4 years	Supervisors
5.	P5IDI	29	F	BSc	6 years	Head nurse
6.	P6IDI	37	F	BSc	5 years	Staff nurse
7.	P7IDI	40	M	MSc	6 years	Nursing director (matron)
8.	P8IDI	33	M	BSc	7years	Head nurse
9.	P9IDI	30	M	BSc	6 years	Head nurse
10.	P10IDI	29	M	BSc	4 years	Supervisor
11.	P11IDI	34	F	BSc	6 years	Nursing director (matron)
12.	P12IDI	30	M	BSc	5 years	Head nurse

The socio-demographic characteristics of the study participants are as follows: a total of 12 individuals participated, including five women and seven men, with ages varying between 29 and 40 years. Among the participants, nine held bachelor’s degrees, while three possessed master's degrees. The professional roles included four nursing directors (matrons), two supervisors, four head nurses, and two staff nurses. Additionally, the participants had between 4 and 8 years of experience working in the nursing field within public hospitals in the Wolaita Zone (see Table 3).

### Organizational factors

#### Type of institution

**Figure 1:**
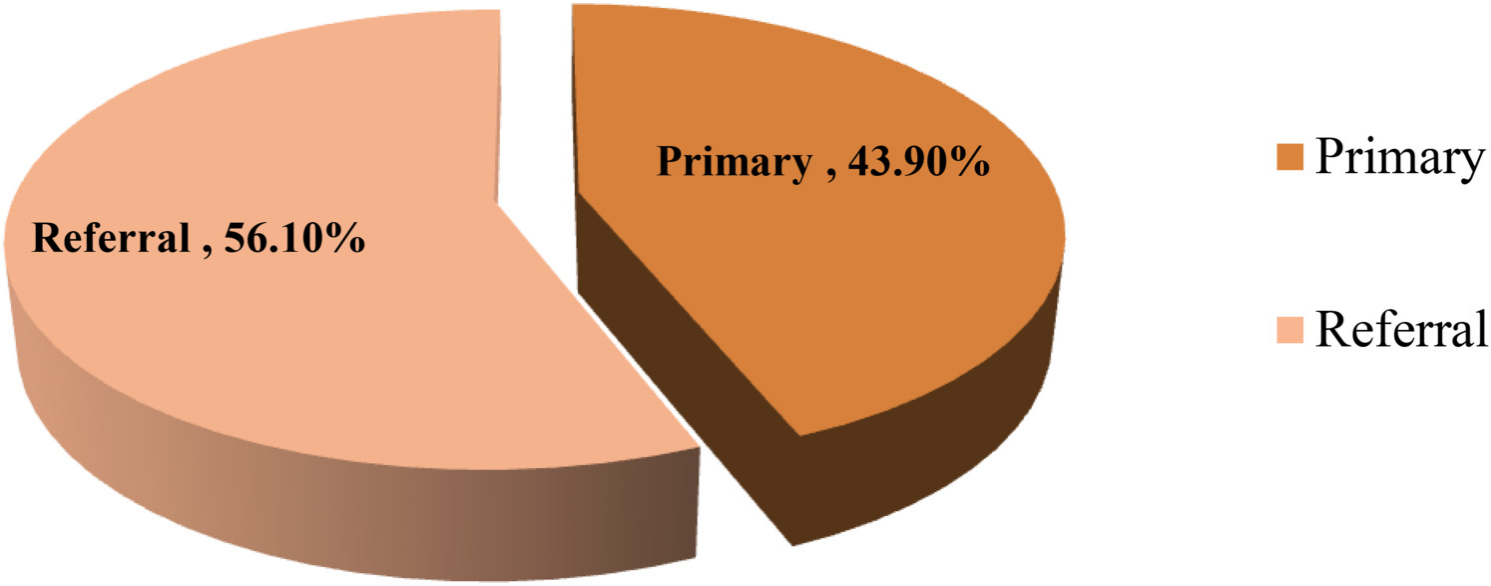
Distribution of nurses according to the level of public hospitals in the Wolaita Zone, southern Ethiopia, August 2023.

The current study found that more than half (56.1%) of nurses were working in a referral hospital, while the rest of the participants were employed at primary hospitals (see Figure 1).

#### Training and skill

**Figure 2:**
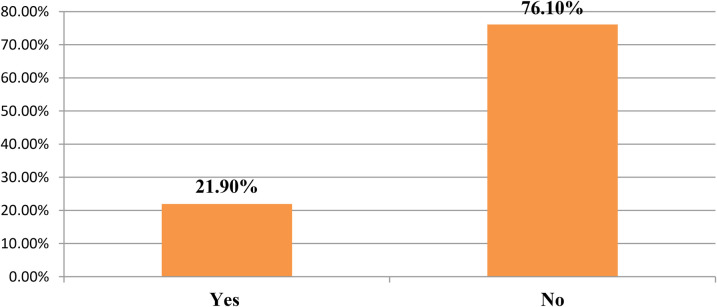
Training and skill among nurses working in public hospitals in the Wolaita Zone southern Ethiopia, August 2023 (*n* = 394).

This study showed that approximately two-thirds of respondents, 291 (73.9%), had not received any training in the past year (see Figure 2). This finding was also supported by responses from IDP participants, who emphasized that continuous professional training is essential for improving nurse's knowledge and skills, advancing the profession, and boosting motivation. For instance, the qualitative findings supported this perspective:

“…In my opinion, nurses’ work motivation can be increased or improved not only by one specific thing, but it is multi-dimensional,…eemmm… for example, it needs a consistent vocational training, however, in our hospital this was only practiced and limited to certain parts of the working unit. Therefore, it is good that continuous professional training should be offered to nurses working in each department or unit…” (33-year-old female, P1IDI)

Another IDI participant said that there was provision of training for nurses working in certain departments:

“…Previously, even once a year, we used to provide training for nurses from some working units. But now due to lack of budget and other related reasons we face difficulty in providing and giving enough training for our nurses …” (40-year-old male, P7IDI)

### Perceived leadership in hospitals

**Figure 3:**
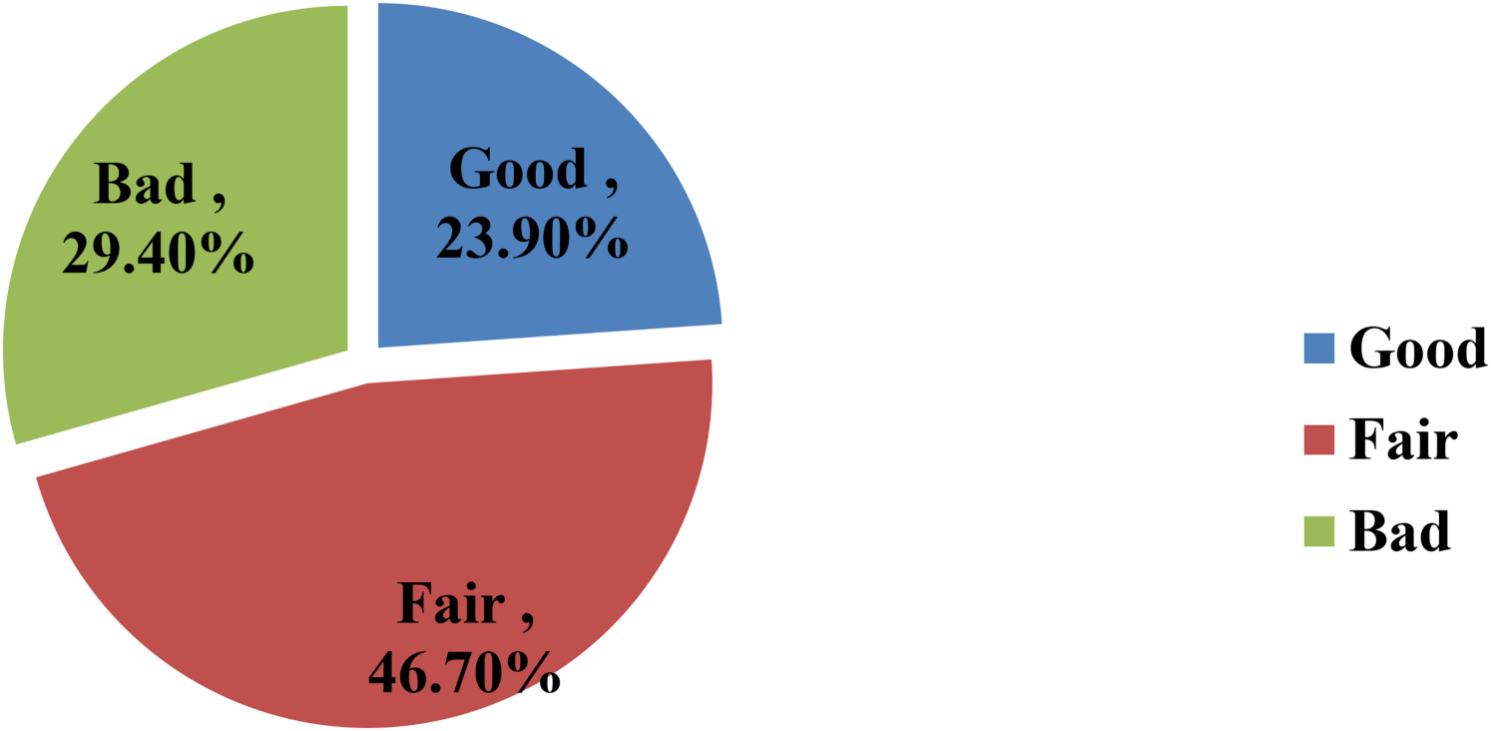
Perceived leadership in hospitals among nurses working in public hospitals in the Wolaita Zone, southern Ethiopia, August 2023 (*n* = 394).

The finding of this study indicated that the majority of respondents (46.7%) rated perceived leadership in hospitals as fair, while the 29.4% perceived it as poor and 23.9% rated it as good (see Figure 3). This finding was further supported by insights from IDI participants, who emphasized the need for focus on improving the leadership to achieve better outcomes of hospital goals. For instance, the findings from a qualitative study also supported this finding:

“…In my understanding one of the many challenges and the root cause for decreased nurses’ work motivation is when there is lack of better administration and leadership in the hospital, which can provide support, quick solution, and fixes problems for questions that arise from nurses. I think we need to improve it more than we did previously…” (32-year-old male, P4IDI)

The other participant added that:

“…For example, sometimes nurses are not happy and disagree with the leadership of the head nurse of the working unit and even they may not be happy with the nurse directors too. As a result, it is difficult to say that these nurses are happy with their work and motivated. So that in such a condition something like this needs to be addressed and solved quickly…” (29-year-old female, P5IDI)

### Respect for nurses in hospitals

**Figure 4:**
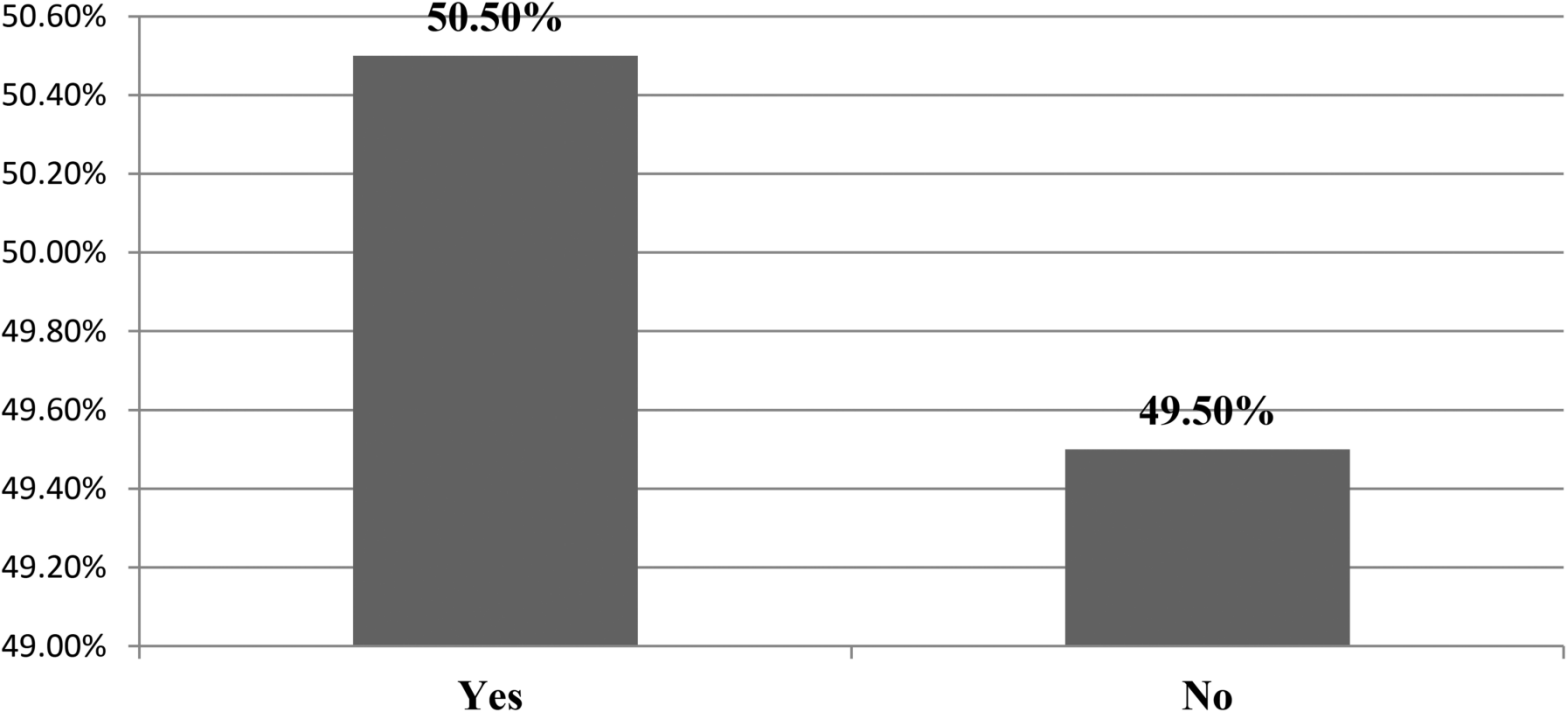
Respect for the nursing profession in public hospitals in the Wolaita Zone, southern Ethiopia, August 2023 (*n* = 394).

This study revealed that more than half of the participants, 199 (50.5%), agreed that the nursing profession is respected by their organization and by other professionals (see Figure 4). Regarding respect for the nursing profession, some participants during an in-depth interview expressed differing ideas, focusing on the concept of respect to nurses, and suggested that the nursing profession is not sufficiently respected and needs improvement at both institutional and community levels. For instance, the qualitative findings supported this result:

“…first of all if a nurse wants to be motivated that nurse should be given love and respect for his own profession, otherwise the other body and professionals will not value his profession. A nurse who loves and respects his profession is motivated to do a better job…” (36-year-old male, P3IDI)

Additionally, an in-depth interview participant indicated that the work motivation of nurses improves when the nursing profession is positively perceived by the community.

“…Furthermore, what I understand from my previous experience and what is common is that the perception of the nursing profession in the community needs to be improved. Nurses should be respected in both the community and the working environment. In that case, nurses will love their profession and be motivated for their job…” (36-year-old male, P3IDI)

### Perceived benefits and remunerations

This study indicated that 163 participants (41.1%) rated the overall response toward the perceived nurses' responsibility in providing patient care, relative to the benefits they receive from their organizations, as good. Additionally, 186 participants (47.2%) perceived the respect and benefits afforded to nurses within their organization as good (see Table 4). According to responses from IDI participants, benefits and other financial incentives are currently a major concern for nurses in each hospital. For instance, the qualitative findings supported this result:

**Table 4:** Result showing perceived benefits and remunerations of nurses working in public hospitals in the Wolaita Zone, southern Ethiopia, August 2023 (*n* = 394).

Variables	Response	Frequency	Percentage
Perceived nurses' responsibility in providing patient care compared to the benefits they received	Good	163	41.4
	Fair	130	33
	Bad	101	25.6
Perceived respect and benefit for nurses in the organization	Good	186	47.2
	Fair	114	28.9
	Bad	94	23.9

“…In my experience, many times nurses want to work in a place where they are paid better or extra overtime (duty). This means that a good payment and benefit is essential for nurses to function happier and motivated toward their work…” (33-year-old male, P8IDI)

One participant's responses supported this finding by stating that nurses are not enough benefited from their hospitals and often seek benefits from other options. For instance, the qualitative findings supported this view:

“…I hope most nurses agree with the idea that the current salary of nurses now paid is disproportionate when compared with the work that nurses do. So many nurses in our hospital do extra work at a private hospital and clinic for extra hours to get better payment …” (30-year-old male, P12IDI)

### Continuous professional development/advancement in job

**Figure 5:**
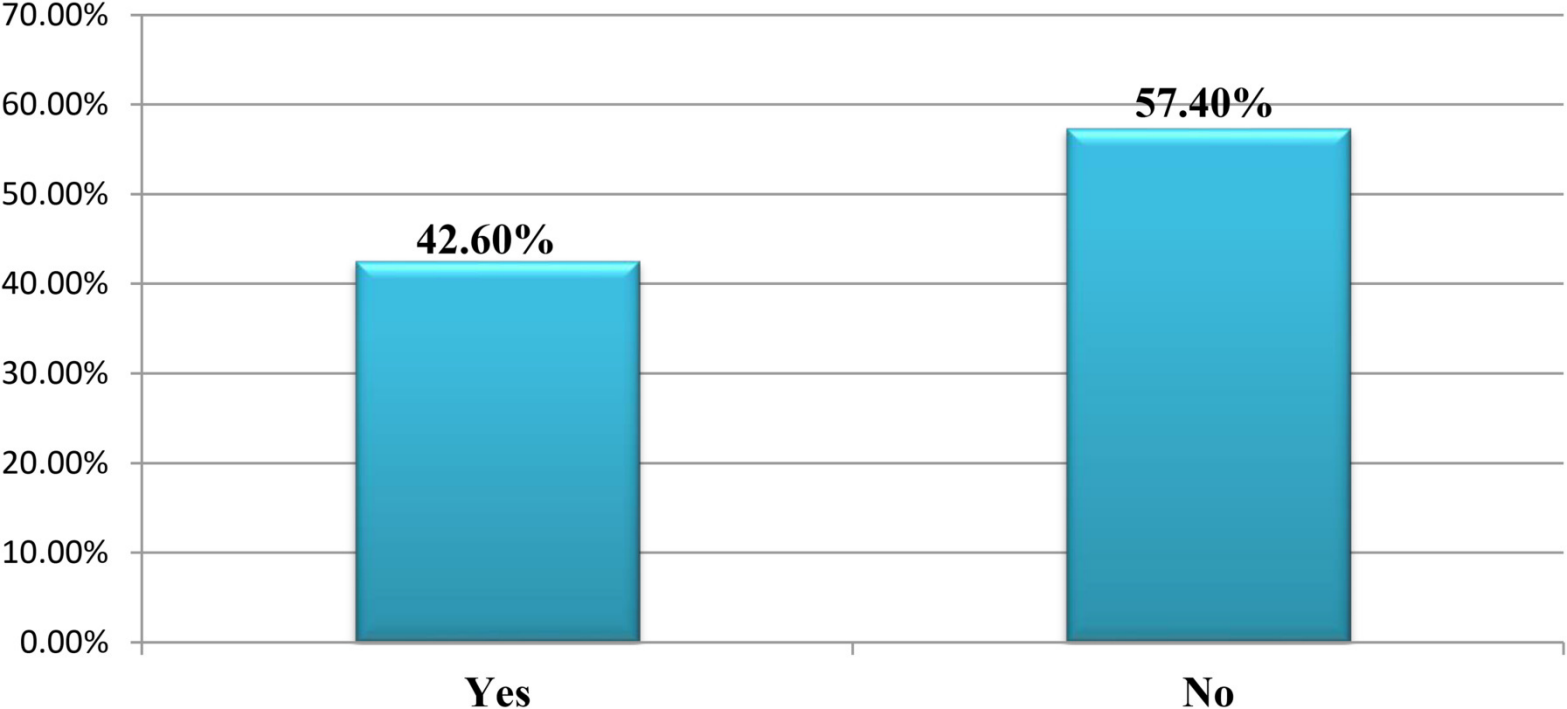
Continuous professional development/advancement in job among nurses working in public hospitals in the Wolaita Zone, southern Ethiopia, August 2023, G.C. (*n* = 394).

This study showed that the majority of the nurse participants, 226 (57.4%), reported not having received continuous professional development in the past year (see Figure 5).

**Figure 6:**
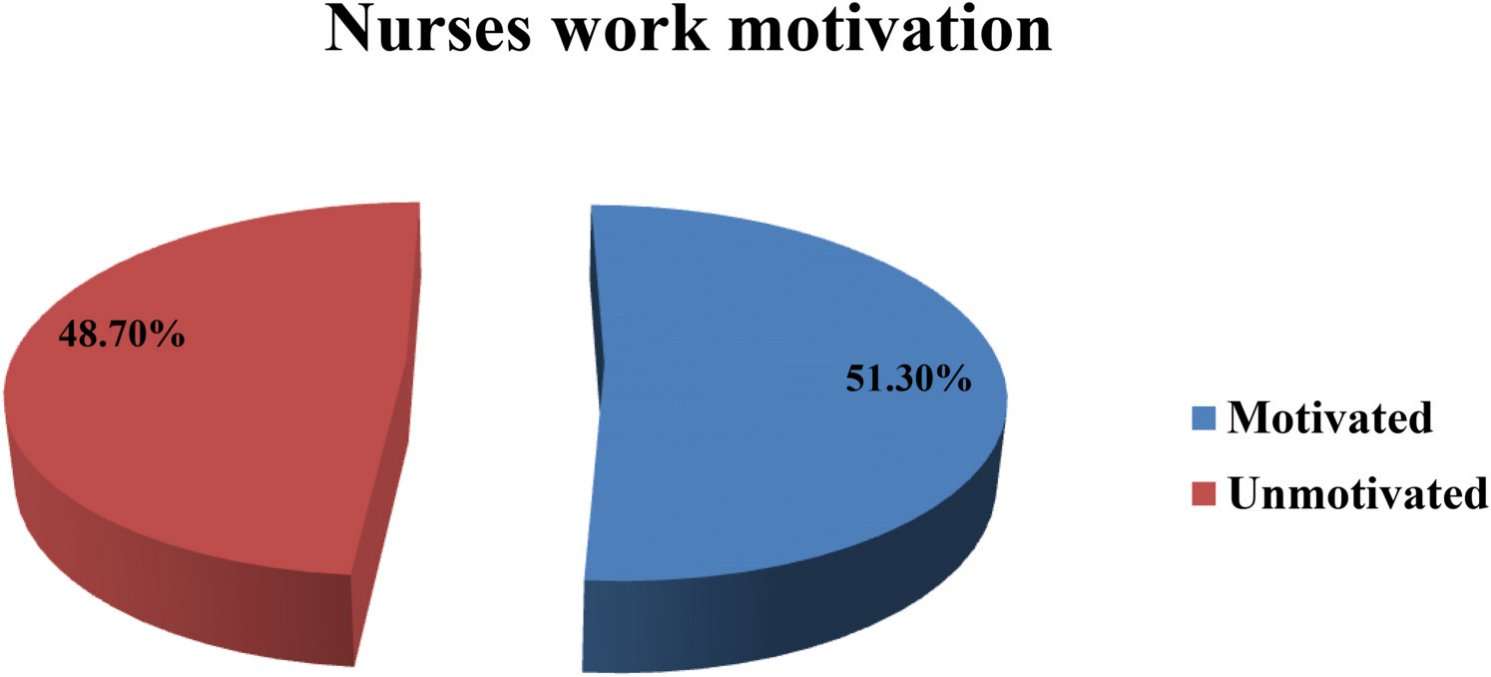
Overall nurses’ work motivation in public hospitals in the Wolaita Zone, southern Ethiopia, 2023 (*n* = 394).

### Work environment-related factor

Numerous factors within the work environment influence nurses’ work motivation, including inter-professional communication, workload, and resource availability. The findings of this study indicated that 168 participants, representing 42.6%, expressed a need for enhancements in inter-professional communication within their hospital setting. Additionally, workload emerged as a significant factor impacting nurses’ motivation, with 221 respondents (56.1%) reporting satisfaction with their current workload. In terms of resource availability, a substantial majority, 283 participants (71.8%), indicated a lack of adequate necessary resources in their workplace (see Table 5).

**Table 5:** Work environment-related factors among nurses working in public hospitals in the Wolaita Zone, southern Ethiopia, August 2023 (*n*=394).

Variable items	Response	Frequency	Percentage
Necessary resources and supplies are available at your institution/hospital	Yes	283	71.8
	No	111	28.2
	No	226	57.4
How did you see the workload in your working unit?	Happy	221	56.1
	Overworked	173	43.9
How did you perceive inter-professional communication in your institution?	Very satisfied	78	19.8
	Not Satisfied	168	42.6
	Need improvement	148	37.6

Regarding resource availability in hospitals, IDI participants emphasized that resources include not only material supplies but also non-material aspects, such as human resources. Availability of necessary medical equipment in hospitals is essential for delivering quality nursing care to patients. For instance, the qualitative findings supported this:

“…Sufficient medical devices along with adequate medication supply and human power in the working unit were necessary to provide better nursing care and performance. Otherwise, nurses will have difficulty doing their jobs properly and they will be demotivated…” (A 34-yrs-old female, P2IDI)

Another study participant reported that there is currently a lack of necessary medication supplies in the hospital, which may influence nurses’ work motivation in various ways. For instance, the qualitative findings supported this view:

“…Currently there is a lack of medications and other medical supplies, which are not available in the hospital and generally on a national level. This may hinder patients’ satisfaction and finally result in poor nursing care provision and treatment. So essential medication and supplies must be adequately supplemented to the nurses to enhance their motivation…” (33-year-old male, P8IDI)

The findings of this study indicated about approximately half of the respondents (51.3%; 95% CI: 46.4–56.9) were classified as motivated, and the mean total motivation score, based on a maximum possible score of 170, was 117.43 (±20.24 SD) (mean = 3.45, median = 3.47, SD = ±0.59). The highest and lowest motivation scores recorded among participants from the overall study were 160 and 39, respectively (see Table 6). The overall motivated nurses were 51.3% while th unmotivated nurses were 48.7% (see Figure 6).

**Table 6:** Work motivation scale of nurses working in public hospitals in the Wolaita Zone, southern Ethiopia, August 2023 (*n* = 394).

Item	Frequency (%)				
	Strongly agree	Agree	Uncertain	Disagree	Strongly disagree
Satisfaction item					
Motivated to work hard	38 (9.6)	107 (27.2)	126 (32.0)	78 (19.8)	45 (11.4)
Satisfied with work	55 (14)	156 (39.6)	104 (26.4)	56 (14.2)	23 (5.8)
Work gives personal achievement	65 (16.5)	189 (48)	81 (20.6)	49 (12.4)	10 (2.5)
Proud to work in this organization	61 (15.5)	152 (3.6)	104 (26.4)	60 (15.2)	17 (4.3)
Benefit items					
Good employment benefits	73 (18.5)	135 (34.3)	96 (24.4)	65 (16.5)	25 (6.3)
Satisfied with the salary	64 (16.2)	114 (28.9)	94 (23.9)	80 (20.3)	42 (10.7)
Payment should be according to experience	75 (19)	124 (31.5)	105 (26.6)	64 (16.2)	26 (6.6)
I work only to get paid	82 (20.8)	117 (29.7)	90 (22.8)	60 (15.2)	45 (11.4)
Recognition item					
I feel that the organization values my work	82 (20.8)	148 (37.6)	89 (22.6)	60 (15.2)	15 (3.8)
I receive recognition for doing good work	86 (21.8)	121 (30.7)	95 (24.1)	69 (17.5)	23 (5.8)
I feel that the community values my work					
Supervision item	97 (24.6)	121 (30.7)	108 (27.4)	54 (13.7)	14 (3.6)
I am satisfied with the supervision	70 (17.8)	133 (33.8)	93 (23.6)	67 (17)	31 (7.9)
Supervisors give regular and timely feedback to help improve the performance	79 (20.1)	102 (25.9)	123 (31.2)	64 (16.2)	26 (6.6)
My supervisor is available when I need support	76 (19.3)	136 (34.5)	95 (24.1)	55 (14)	32 (8.1)
I have a work plan developed with my supervisor	96 (24.4)	120 (30.5)	89 (22.6)	71 (18)	18 (4.6)
Item	Frequency (%)				
	Strongly agree	Agree	Uncertain	Disagree	Strongly disagree
Management items					
There is transparency in performance evaluation in the organization	84 (21.3)	124 (31.5)	98 (24.9)	68 (17.3)	20 (5)
There is a good relationship between management and staff	93 (23.6)	136 (34.5)	85 (21.6)	63 (16)	17 (4.3)
The organizational mission is understood and clear to all	81 (20.6)	124 (31.5)	101 (25.6)	68 (17.3)	20 (5.1)
The promotion criteria in the healthcare institution are clear and understood by all	87 (22.1)	121 (30.7)	109 (27.7)	60 (15.2)	17 (4.3)
There is equal treatment among co-workers	91 (23.1)	113 (28.7)	103 (6.1)	62 (15.7)	25 (6.3)
The organization provides us with skill and knowledge	87 (22.1)	138 (35)	93 (23.6)	59 (15)	17 (4.3)
This health facility inspires us to do our very best on the job	78 (19.8)	147 (37.3)	95 (24.1)	58 (14.7)	16 (4.1)
Work arrangements and the performance standards expected from staff are clear and understood by all	74 (19.3)	130 (33)	98 (24.9)	64 (16.2)	26 (6.6)
There is an environment of co-operation between staff and management	66 (16.8)	128 (32.5)	106 (26.9)	71 (18)	23 (5.8)
There is availability of tools and materials for skillfuluse	70 (17.8)	125 (31.7)	101 (25.6)	71 (18)	27 (6.9)
There is respect and trust from clients	86 (21.8)	145 (36.8)	87 (22.1)	62 (15.7)	14 (3.6)
Goal clarity					
Job, duty, and requirements are specific and clear	77 (19.5)	125 (31.7)	110 (27.9)	62 (15.7)	20 (5.1)
There is a clear objective to achieve	86 (21.8)	129 (32.7)	109 (27.7)	58 (14.7)	12 (3)
There is no interference in the job	70 (17.8)	142 (36)	98 (24.9)	73 (18.5)	11 (2.8)
Satisfied to use ability in job	77 (19.5)	147 (37.3)	100 (25.4)	56 (14.2)	14 (3.6)
Opportunity					
Good opportunity to continue education	92 (23.4)	126 (32)	95 (24.1)	67 (17)	14 (3.6)
Adequate in-service training to address the skill gap	82 (20.8)	119 (30.2)	88 (22.3)	85 (21.6)	20 (5.1)
You prefer not to continue working in this organization	92 (23.4)	117 (29.7)	86 (21.8)	79 (20.1)	20 (5.1)
As soon as you find a better job, you quit working in this organization	84 (21.3)	138 (35)n	93 (23.6)	60 (15.2)	19 (4.8)
Nurses’ work motivation	Motivated		202 (51.3%)		
	Unmotivated		192 (48.7%)		

### Factors associated with nurses’ work motivation

The bivariate analysis identified 11 variables with a *p*-value less than or equal to 0.25 . Sex, work experience, qualification, monthly benefit, type of institution, perceived respect and benefits for nurses within the organization, perceived management and leadership of the organization, training, workload, inter-professional collaboration, and resource availability were identified as candidate variables for multivariable logistic analysis. All candidate variables were entered into a multivariable logistic regression model using a backward likelihood ratio method to determine the final predictors of nurses’ work motivation while controlling for potential confounders.

In the multivariable logistic regression, five variables were found to be the statistically significant predictors of nurses’ work motivation (*p*-value < 0.05, 95% CI).These included type of institution, workload, perceived respect and benefit, perceived leadership in the hospital, and work experience, all of which were found to be independent predictors of nurses’ work motivation.

Nurses working in referral hospitals were 59.4% less likely to be motivated than those working in primary hospitals (AOR = 0.424; 95% CI: 0.236–0.699; *p* = 0.033) (see Table 7). For instance, this finding is supported by the qualitative data:

**Table 7:** Bivariate and multivariable logistic regression showing factors affecting work motivation among nurses working in public hospitals in the Wolaita Zone, southern Ethiopia, August 2023.

Variables	Category	Nurses’ work motivation		COR (95%CI)	AOR (95%CI)	*p*-Value
		Motivated *n* (%)	Unmotivated *n* (%)			
Type of institution	Pr.Hos	117 (67.6)	56 (32.4)	1	1	
	Ref.Hos	85 (38.5)	136 (61.5)	0.299 (0.197,0.454)*	0.406 (0.236,0.699)	**0.001**
Experience	1–5 years	157 (72.4)	60 (27.6)	4.486 (1.686,11,934)*	3.29 (1.101,9.846)**	**0.033**
	6–10 years	38 (24.1)	120 (75.9)	0.54 (0.20,1.477)	0.555 (0.180,1.707)	0.304
	>11 years	7 (36.8)	12 (63.2)	1	1	
Workload	Happy	142 (64.5)	78 (35.5)	1	1	**.007**
	Overworked	60 (34.5)	114 (65.5)	0.289 (0.190, 0.439)*	0.495 (0.297,0.827)**	
Perceived respect and benefits for nurses	Good	118 (63.4)	68 (36.6)	1	1	
	Fair	59 (51.8)	55 (48.2)	0.618 (0.385,0.992)	0.841 (0.485,1.460)	0.539
	Bad	25 (26.6)	69 (73.4)	0.209 (0.121,0.361)*	0.351 (0.180,0.682)**	**0.002**
Perceived leadership in hospitals	Good	59 (53.2)	52 (46.8)	1	1	
	Fair	107 (58.8)	75 (41.2)	1.257 (0.782,2.023)	1.076 (0.598,1.937)	0.807
	Bad	36 (35.6)	65 (64.4)	0.488 (0.281,0.848)*	0.487(0.240,0.985)**	**0.045**

Pr.Hos, primary hospitals; Ref.Hos, referral hospitals.

* (*P* < 0.25) in bivariate; 1, reference group; **Statistically significant in multivariables.

The bold values are the *p*-value of significantly associated variables.

“…working in referral hospital needs internal commitment regardless of personal issues. Nowadays I am very surprised when I think about the motivation of nurses. I think that it seems most of the nurses do not like their profession, including me. Currently it is difficult to say that there is a good motivation for the work of nurses, so attention should be paid to improve motivation of nurses” (36-year-old female nurse, P3IDI)

“…In general nurses working in referral hospitals should be motivated well enough because most of patient came for medical cares are referral cases and those with advanced disease. So in current condition what I have been experienced and observed in the past years, the work motivation of nurses needs improvement in our hospital. …” (37-year-old female, P6ID)

Regarding work experience, nurses with 1–5 years of work experience were 3.29 times more likely to be motivated than those with more than 5 years of experience (AOR = 3.29; 95% CI: 1.101–9.846; *p* = 0.033) (see Table 7).

For instance, this finding is also supported by qualitative in-depth interview data:

“…In our work unit there is a lot of work to be done that makes us a special unit from other work units. Because in our unit, nursing work requires strict care and precaution, for instance, when we do a nursing shift or round every six months with our nursing management, we do assign nurses with good working behavior and those who have not been employed for long. Because we expect those who haven't been employed for longer are ready for new challenges; they accept our advice; these nurses are often able to know and learn, and their readiness and boredom levels are low.” (29-year-old female, P10IDI)

“…What I've seen from my work experience and what I'm still doing is that when nurses start work here in our unit or are hired here, I'll assign them for a couple of weeks with other experienced nurses, but gradually then I see them they are more confident on their activity. However, not all of these nurses, but most of them have a good tendency to capture and learn new things and they are motivated to work.” (34-year-old female, P1IDI)

Concerning workload, nurses who reported being overworked in their units were 50.5% less likely to be motivated than those who were happy with their workload (AOR = 0.495; 95% CI: 0.297–0.827; *p* = 0.007) (see Table 7). For instance, this result is supported by qualitative in-depth interview data:

“…even though there are enough nurses in number in our hospital, we see that nurses are busy throughout day and night (even for three shifts) due to workloads. In many work units patient flow is not balanced with the resources available. Because a lot of patients come from the nearest zone or regions, district hospitals and health centers with referral cases, because it'*s the only referral hospital in our zone, so I hope the workload will decrease if an additional nurses will be hired in future and even constructing one more general hospital in zonal level.”* (32-year-old male, P4IDI)

Nurses who had a negative perception of respect and benefits for nurses in their organization were 64.9% less likely to be motivated than those with a positive perception (AOR = 0.351; 95% CI: 0.180–0.682; *p* = 0.002) (see Table 7). For instance, this finding is supported by the qualitative data:

“…I'm always trying to bring up the point as much as possible when I get a chance. eemm…In my opinion and what I experienced nurses should be respected as any health care provider, but I don't usually get much of a response or see any attempts to address the complaints made by nurses at our hospital. It seems like the hospital or concerned body does not really make an effort to show respect for nurses as professionals.” (40-year-old female, P7IDI)

“…In terms of the number of nurses working in our hospital, most nurses have good work motivation. However, due to multiple reasons, for example, part-time work incentives, or overtime payments have been delayed for several months without payment (**wuzif**). The other reason is also risk payment (risk allowance) is not paid for those nurses working in risky units, while it is paid for others working in other similar hospitals in our woreda. The average overtime hour per month is not similar in each working unit, so I hope there will be good nursing initiatives and motivation if the reasons i have been raised above are corrected.” (30-year-old male, P9IDI)

Perceived leadership in hospitals was another factor associated with nurses’ work motivation. The findings indicated that nurses who had a negative perception of leadership were 51.3% less likely to be motivated than those who had a positive perception (AOR = 0.487; 95% CI: 0.240–0.985; *p* = 0.045) (see Tables 7, 8**)**. For instance, this finding is supported by the qualitative data:

**Table 8:** Theme and sub-themes/categories of qualitative findings among nurses working in public hospitals in the Wolaita Zone, southern Ethiopia, August 2023, G.C. (*n* = 394).

Themes	Sub-themes/categories	Codes
Structural-administrative factors	Leader-related	• Inadequate support • Inadequate provision of basic PPE • Lack of medication and supplies • Valuing nurses or respect • Poor guiding and support • Lack of training
	Work unit-related	• Long working time • Type of hospital • High patient flow • Care fatigue • Conflict in units • Poor patient to nurse communication • Poor nurse to nurse communication
Socio-economic factors	Inadequate salaries	• Less salaries • Delayed payment • Working for an additional income source
	Extra time payment	• Delayed payment
	Lack of additional incentives	• Lack of house allowance • Lack of transportation allowance
Individual nurse-related factors	Respecting own profession	• Poor image for own profession
	Professional ethics and conduct	• Absenteeism from work • Poor time management
	Need of support	• Family support • Friends’ or co-workers’ support • Community support

“…Nurses stay or spend more hours and spend longer with patients than any other health care professional, so whatever they feel discomfort regarding service provision or related to nursing care or even the need for necessary resource supplies, the managers and administrators should listen to the nurses and provide support. These activities are not regularly practiced in our hospital. Therefore, I recommend that appropriate action should be taken when such a problem arises…” (34-year-old female, P2IDI)

Another 34-year-old female head nurse stated that

“…I often think that the relationship or communication between managers/leaders at any level or with the person in charge and staff nurses is not clear and open. For instance, those responsible persons who lead nursing departments in hospitals, even at the school level or nationally (ENA) should be committed to the profession and make clear information regarding the profession. Those responsible people should talk openly with experts if there is an administrative problem, but if it is not resolved in time, the consequences will result in patients, institutions, and eventually affect the local area and the country as a whole.” (33-year-old male, P8IDI)

Work motivation of nurses can be influenced by the type of institution, work experience, workload, perceived lack of respect, inadequate benefits, and poor leadership. Regarding the qualitative finding, three major themes emerged: structural-administrative factors, socio-economic factors, and individual nurse-related factors, each with their respective sub-themes/categories and codes.

## Discussion

The current study revealed that the overall level of nurses’ work motivation was 51.3% (95% CI: 46.4–56.9), based on scores above the standardized mean, which indicates that the nurses were motivated. However, this also showed that nearly half of the study participants were unmotivated in their work, which may lead to client dissatisfaction, poor quality of nursing care, increased burnout, and decreased commitment to the institution.

The findings of the current study are consistent with the studies conducted in Iran, Vietnam (53.4%), and Jimma Town, where 55.5%, 53.4%, and 54.5% of nurses were motivated, respectively (26, 41, 42). In contrast with the above findings, the result of the current study is inconsistent, and the motivation level in this study is higher than that reported in studies conducted in Bulgaria (40.3%), Cameroon (42.7%), Jimma (25.1%), and Gedio Zone, where only 19.5% of the study participants were motivated (12, 22, 43, 44). This discrepancy is mainly due to variations in different aspects of the condition. For instance, the health system structures in foreign countries differ from that of Ethiopia. Another possible explanation relates to differences in study settings and time periods; all three studies, except for the one conducted in Bulgaria, were conducted 6 years ago, while the current study was conducted more recently, during a time when there may have been some policy changes and improvements in the health system, which could account for this discrepancy. Additionally, the studies conducted in the Gedio Zone and Jimma included health professionals, while this study considered only nurses as study participants. Moreover, since the current study used a self-administered questionnaire, there is a possibility of over-reporting by the participants, which may have resulted in increased levels of motivation in the study area.

However, the findings of the current study are lower than those reported in studies carried out in the West Arsi Zone, West Amhara, and Central Ethiopia, where the overall motivation levels were 58.3%, 58.6%, and 63.63%, respectively (23–25). This inconsistency might be due to differences in study settings. For example, the study in the West Arsi Zone was conducted across health centers in seven districts, while the current study included all hospitals within the study area.

This research was conducted in a multicenter setting within Central Amhara, encompassing three hospitals, and included all nine public hospitals in the region. In contrast, previous studies conducted in West Amhara and Central Ethiopia focused on participants such as doctors, anesthetists, and pharmacists, who generally enjoy a relatively favorable socio-economic status, particularly in terms of salary and benefits. The current study, however, exclusively involved nurses, which may affect the comparative analysis of motivation levels among different professional groups. Additionally, work motivation is subject to fluctuations over time and varies between institutions, leading to notable differences in motivation levels observed across various regions (27).

The results of this study suggest that the type of healthcare institution significantly affects nurses’ work motivation. Specifically, nurses employed in referral hospitals exhibited a 59.4% lower likelihood of being motivated compared to their counterparts in primary hospitals. This finding aligns with research conducted in Jimma Town, Ethiopia, which indicated that nurses working in referral hospitals were 83.8% less likely to be motivated than those working in primary hospitals and health centers (26). However, another study throughout the health facility found no significant association between nurses’ motivation and the type of institution in which they worked (38).

In contrast to this finding, a cross-sectional study conducted in West Amhara revealed that healthcare professionals from primary hospitals had, on average, 0.143 units lower intrinsic motivation scores compared to those from referral hospitals (23). This discrepancy may be attributed to several factors, including the smaller sample size used in that study (304 participants), differences in study units or study areas, as it included various health professionals rather than focusing exclusively on nurses, variations in the time and year the study was conducted. Another possible explanation for this could be that, in the current study, more than half of the participants were from referral hospitals, which may have affected the study results

Additionally, referral hospitals have many departments, extended working hours, and multiple workplace factors that can affect nurses’ motivation. Hence, this is supported by studies conducted in several referral hospitals in Ethiopia, which found that nurses perceived that their work environment as unhealthy, especially in terms of participation in hospital affairs, management support, and involvement in quality of care (9, 45). Another possible explanation is that workplace stress negatively influences work motivation; this is supported by a study conducted at Felege Hiwot referral hospital in Bahir Dar, which reported a high prevalence of workplace stress (46). Such stress, in turn, negatively affects nurses’ work motivation.

Work experience was identified as a significant factor influencing nurses’ work motivation; nurses with 1–5 years of experience were 3.29 times more likely to be motivated than those with more years of experience. This finding is consistent with a study conducted in Turkey among nurses and midwives. A study in Jimma Town, Ethiopia, also supports this finding, revealing that more experienced nurses were 65.7% less likely to be motivated than their less experienced counterparts (26, 47). A possible explanation for this may be that as the number of working years increases, nurses experience a decline in job satisfaction and motivation, potentially due to growing family responsibilities and an inability to derive necessary satisfaction and motivation from their work.

The current result is not consistent with studies conducted in other parts of the world, such as Zambia, Greece, and Germany, which suggested that nurses with more years of experience tend to exhibit higher levels of job motivation compared to their less experienced counterparts (33, 48–50). This discrepancy may be attributed to the fact that nurses with more years of experience or a higher self-rated expertise may experience a sharp increase in work motivation (20).

Regarding workload as an influencing factor of nurses’ work motivation, nurses’ who reported being overworked in their units were 50.5% less likely to be motivated than those who were happy with their workload. This finding aligns with the studies conducted in Kenya, Bulgaria, Vietnam, and the Gedeo Zone, which revealed a negative association between workload and work motivation (12, 22, 51, 52). This might be because a high workload significantly influences nurses’ work motivation; a high workload can lead to fatigue, reducing nurses’ ability to focus on tasks and ultimately resulting in decreased work motivation (53).

Perceived respect and benefits within an organization are also key factors influencing nurses’ work motivation. Nurses who had a negative perception of the respect and benefit within their organization were 64.9% less likely to be motivated than those with a positive perception. This finding is supported by a qualitative study conducted among Croatian nurses, which reported that dissatisfaction among nurses was mainly due to poor relationships with superior managers and a general lack of appreciation and respect for the nursing profession (54). The study conducted in Cameroon also supports this finding (55). A possible explanation is that nurses require appreciation and recognization to fulfill their needs for reputation, prestige, and respect from others, which eventually can increase their self-esteem.

Studies conducted in Pakistan, Saudi Arabia, and Bulgaria have revealed that salary and financial benefits are the most significant factors positively influencing nurses’ work motivation (12, 18, 19, 22, 25, 26, 48, 56). A study conducted in Addis Ababa, Ethiopia, also supports this finding, indicating that salary and benefits are the most important and influential variables (57). A possible explanation for this is that income and financial allowances are powerful motivators capable of fulfilling various individual needs. Moreover, receiving a good salary can foster a feeling of enjoyment and pleasure in one’s work.

In contrast with the current finding, a study conducted in India revealed that benefits and remunerations had the least influence on the work motivation of frontline employees (20). A possible explanation for this discrepancy may lie in differing values and wants of nurses; in the Indian context, nurses may place great emphasis on meeting their internal motivating factors, like participation in independent decision-making, which are considered important drivers of motivation within healthcare organizations.

Perceived leadership in hospitals was also identified as a significant factor associated with nurses’ motivation. Nurses who perceived leadership as poor were 51.3% less likely to be motivated than those who perceived leadership positively. This finding is supported by a study conducted in Vietnam, which reported that more than 80% of nurses were motivated to work with good leadership and management content (42). A possible explanation for this finding is that good management practices eases communication between hospital management and subordinates, which in turn minimizes personal and organizational problems, which may enhance nurses’ work motivation.

Additionally, a study conducted in Istanbul revealed that nurses stated that their managers adopted a democratic leadership style. Nurses with a democratic leadership style trust their subordinates and encourage employee participation in decision-making (58). This leadership style is followed by leaders who inspire others, have the vision to elevate employee beliefs, attitudes, and motivation, and create a high-performing workforce (59).

### Strengths and limitations

This study employed a multicentered approach by including all public hospitals within the Wolaita Zone, thereby enhancing its generalizability. Additionally, the qualitative component of the research was executed and documented in accordance with the three domains outlined in the Consolidated Criteria for Reporting Qualitative Research (COREQ) checklist. Nonetheless, the questionnaire utilized was susceptible to social desirability bias, as it relied on self-reported data, which may have led to an overestimation of certain behaviors. Furthermore, due to the cross-sectional design of the study, establishing a cause-and-effect relationship was not feasible. We did not incorporate established theories such as Herzberg's two-factor theory, Maslow's hierarchy of needs, or the self-determination theory.

### Implications for nursing practice

The implications of nurses’ work motivation for nursing practice are profound and manifested in several dimensions. As frontline healthcare providers, nurses play a crucial role in delivering quality care within healthcare institutions; thus, fostering their motivation and ensuring job satisfaction are essential. A lack of motivation can hinder nurses’ ability to perform optimally. The implications for nursing practice include enhanced patient care, improved work performance, greater job satisfaction, decreased turnover rates, better teamwork, and increased adaptability to change. Consequently, it is vital to foster and sustain these implications by promoting nurses’ work motivation.

## Conclusions and recommendations

The findings indicate that nearly half of the participating nurses reported feeling motivated. Work motivation of nurses can be influenced by the type of institution, years of work experience, workload, perceived respect, adequacy of benefits, and the quality of leadership. Regarding the qualitative findings, three major themes emerged, such as structural-administrative factors, socio-economic factors, and individual nurse-related factors. Therefore, hospital administrators must focus on recognizing, training, and promoting nurses to enhance their motivation and overall job satisfaction. Based on the study findings, the following recommendations are suggested:

**Hospital management bodies:**

• Provide opportunities for nurses to enhance their skills and knowledge through continuing education programs, certifying them by providing continuous training, and career advancement.
• Create a positive work environment that promotes collaboration and open communication to timely resolve issues raised by nurses.
• Implement strategies that promote work–life balance, such as flexible scheduling to balance workload by making the working environment safe.
• Acknowledge and appreciate more experienced nurses for their hard work and dedication through regular recognition or public appreciation.
• Hospital administrators of respective hospitals must include clearly defined policies relating to nursing procedures and personnel issues.

**Nursing leaders (supervisors, head nurses, matrons):**

• Encourage supportive supervision by paying more consideration and interest to the feelings of nurses and foster fairness by treating all nursing staff equitably within each institution.
• Clearly define job responsibilities, performance expectations, and goals.
• Work on proactive measures to ensure nurses’ participation in decision-making processes through regular meetings with the nursing staff.
• Encourage nurses to be ethically committed to their work.

**Nursing professionals:**

• Nurses should be aware of their professional ethical values by showing respect to their profession and skills.
• Nurses should strengthen their communication with co-workers and hospital management.
• Nurses should pursue the nursing field for passion and love for the profession rather than solely for other benefits.

**Nurse educators:**

• Should place great emphasis and focus on instilling the values and ethical principles of the nursing professional in their students.

**Researchers:**

• Since the current study was limited to public hospitals only, future research should involve private hospitals for a comparative analysis. This would help in enhancing available literature and offer a better understanding of the motivation level and the factors influencing it across private and public hospitals.

## Data Availability

The original contributions presented in the study are included in the article/Supplementary Material, further inquiries can be directed to the corresponding author.
